# Bottom-Feeders Eat Their Fiber: Ingestion of Anthropogenic Microdebris by Antarctic Deep-Sea Invertebrates Depends on Feeding Ecology

**DOI:** 10.1021/acs.est.4c09487

**Published:** 2024-11-21

**Authors:** Gabriel Stefanelli-Silva, Pâmela Friedemann, Beatriz Rocha de Moraes, Romulo Augusto Ando, Lúcia de Siqueira Campos, Mônica Angélica Varella Petti, Craig R. Smith, Paulo Yukio Gomes Sumida

**Affiliations:** † Departamento de Oceanografia Biológica, Instituto Oceanográfico da Universidade de São Paulo (IO-USP), São Paulo 05508-120, Brazil; ‡ Departamento de Ecologia, Instituto de Biociências da Universidade de São Paulo (IB-USP), São Paulo 05508-090, Brazil; § Departamento de Química Fundamental, Instituto de Química da Universidade de São Paulo (IQ-USP), São Paulo 05509-900, Brazil; ∥ Departamento de Zoologia, Instituto de Biologia da Universidade Federal do Rio de Janeiro (IB-UFRJ), Rio de Janeiro 02141-902, Brazil; ⊥ Department of Oceanography, University of Hawai’i at Ma̅noa, Honolulu, Hawai’i, United States

**Keywords:** microplastics, marine debris, marine benthos, feeding guild, Southern Ocean, biological archives, museum collections

## Abstract

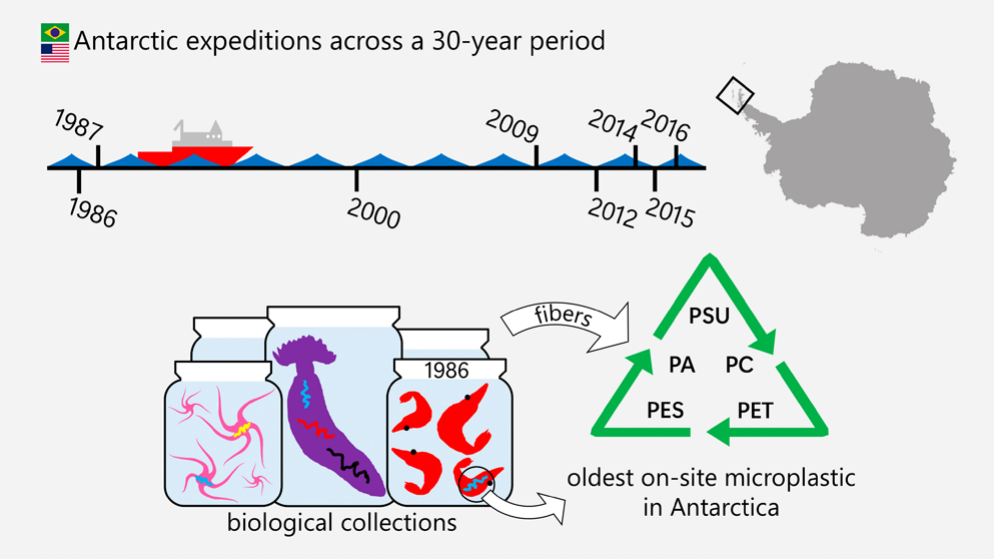

Anthropogenic debris has been documented in Antarctica for the past 40 years. Upon breakdown, large pieces become microdebris, which reaches the seafloor through a variety of physical and biological processes. The Antarctic benthos, deeply reliant on sinking organic particles, is thus vulnerable to ingesting microdebris. By using benthic specimens sampled between 1986 and 2016 and deposited in biological collections, we provide the first record of microdebris in Southern Ocean deep-sea invertebrates. Specimens from 15 species (n = 169 organisms) had their gut content examined, with 13 species yielding microdebris in the shape of fibers (n = 85 fibers). The highest ingestion percentages were recorded in the sea cucumbers *Heterocucumis steineni* (100%), *Molpadia violacea* (83%) and *Scotoplanes globosa* (75%), and in the brittle star *Amphioplus peregrinator* (53%). Deposit- and suspension-feeding were the strategies which yielded the most fibers, accounting for 83.53% of particles. Seven fibers were identified as microplastics, composed of polyamide, polycarbonate, polyester, polyethylene terephthalate, polyisoprene and polysulfone. We also provide the earliest record of a microplastic in Antarctica, a polysulfone fiber ingested by a *Boreomysis* sp. mysid caught in 1986. The occurrence of fibers in the world’s most remote continental margin renews concerns of pollution in seemingly isolated regions.

## Abstract 2

Anthropogenic fibers were ingested by deep-sea megabenthos from different feeding modes and sampled since 1986 in the Antarctic shelf. These findings renew concerns regarding human impacts on this supposedly pristine environment.

## 1 Introduction

Following the current business model of cheap fast fashion, clothing production reached the landmark of 100 billion items in the mid-2010s.^1^ Niinimäki et al. (2020)^2^ estimated an annual release of 2.9 Gt of CO_2_ caused by the production of fibers, textile manufacturing and garment construction – approximately 7.89% of the 36.77 Gt released globally from fossil fuel and industry emissions.^3^ Fibers can be either natural (cotton, silk, wool), synthetic (plastic) or semisynthetic (cellulose-based) and are classified as microdebris^4^ (MD) when smaller than 5 mm, and as microplastics (MPs) when simultaneously <5 mm and synthetic in origin.^5^ The process of commercial and domestic garment washing is one of the reasons behind the release of tiny fibers into the environment, as built-in filters and wastewater treatment plants are not yet equipped to retain such particles.^6,7^ Fibers are found from coasts^8^ to gyres,^9^ and from surface waters^10^ to the deep sea,^11^ across the entirety of the ocean and even in supposedly isolated regions.

Antarctica remains the least explored continent on Earth, but anthropogenic litter – which inevitably will break down into smaller particles −, has been documented washing up on sub-Antarctic islands since the mid-1980s.^12^ While the first record of MD in Antarctic biota dates to 1960, following the ingestion of plastic pellets by dead *Pachyptila* spp. prions, these were obtained from organisms driven ashore New Zealand beaches.^13^ Therefore, on-site research explicitly dedicated to MD in the Southern Ocean only began in the past decade,^14^ owing to a growing interest in the study of plastic distribution in the ocean. Fibers in Antarctica may come from ship and research station wastewater,^15,16^ as well as from atmospheric circulation.^17,18^ Ingestion of fibers has now been reported in a myriad of Antarctic organisms encompassing different feeding guilds and trophic levels: in penguins,^19,20^ seals,^21^ fish,^22^ amphipods,^23^ krill,^24,25^ and the benthic invertebrate community.^26,27^ These occurrences are not only a direct consequence of the growing human footprint in the region, but certain intrinsic characteristics of the Antarctic marine ecosystem may even exacerbate its vulnerability to anthropogenic pollution.

Glacial erosion has resulted in the unusually deep carving (average of 400–500 m)^28,29^ of the shelves surrounding the Antarctic continent. Due to the greater depths in these areas, absence of in situ primary production means that the deep-sea benthos is almost entirely dependent on sinking phytodetritus from the surface ocean.^30^ Free-falling organic matter is made even more crucial to local ecosystem functioning due to the persistence of a seafloor food bank which lasts throughout the low-productivity winter months, likely due to decreased bottom-water temperatures.^31^ The local pelagic-benthic coupling is thus susceptible to the presence of anthropogenic MD, which can also descend through the water column as marine snow,^32^ and via convection,^33^ storm-induced mixing^34^ and termohaline-driven currents.^35^ While fibers that reach the deep sea could remain there indefinitely,^11,36^ due to logistical and financial constraints^37−39^ the impacts on the local fauna are still poorly understood when compared to records in shallow-water benthic organisms.^10,40−45^

In the age of fast-paced environmental change, long-term biological archives are being increasingly used to help understand ecological questions pertaining to anthropogenic impacts.^46^ Research using Antarctic fauna archives includes the characterization of the continent’s deep-sea biodiversity,^28,47,48^ the investigation of how primary production modulates growth rates in bryozoans,^49^ and the determination of the region’s paleoclimate through past ecological changes (reviewed in Strugnell et al., 2022^50^). However, the number of studies which have approached archived materials to verify the presence of MPs in historical samples is still growing, mainly due to the possibility of contamination during past handling. Considering this caveat, however, the examination of particles obtained from inside the guts of intact specimens is a possible way to remedy methodological limitations of decades-old specimens, as effectively done by Courtene-Jones et al. (2019).^51^

So given the historical sparseness of data from deep remote environments in the Southern Hemisphere,^52^ especially regarding the Southern Ocean, our aims in this paper were to (i) detect anthropogenic MD in deep-sea sediment-dwelling invertebrates from Antarctica, (ii) determine whether specimens preserved in biological collections could provide the oldest record of MD in this region, and (iii) verify whether the number of ingested particles was influenced by the feeding mode and size of the studied organisms.

## 2 Materials and Methods

### 2.1 Study Area and Field Work

**Figure 1:**
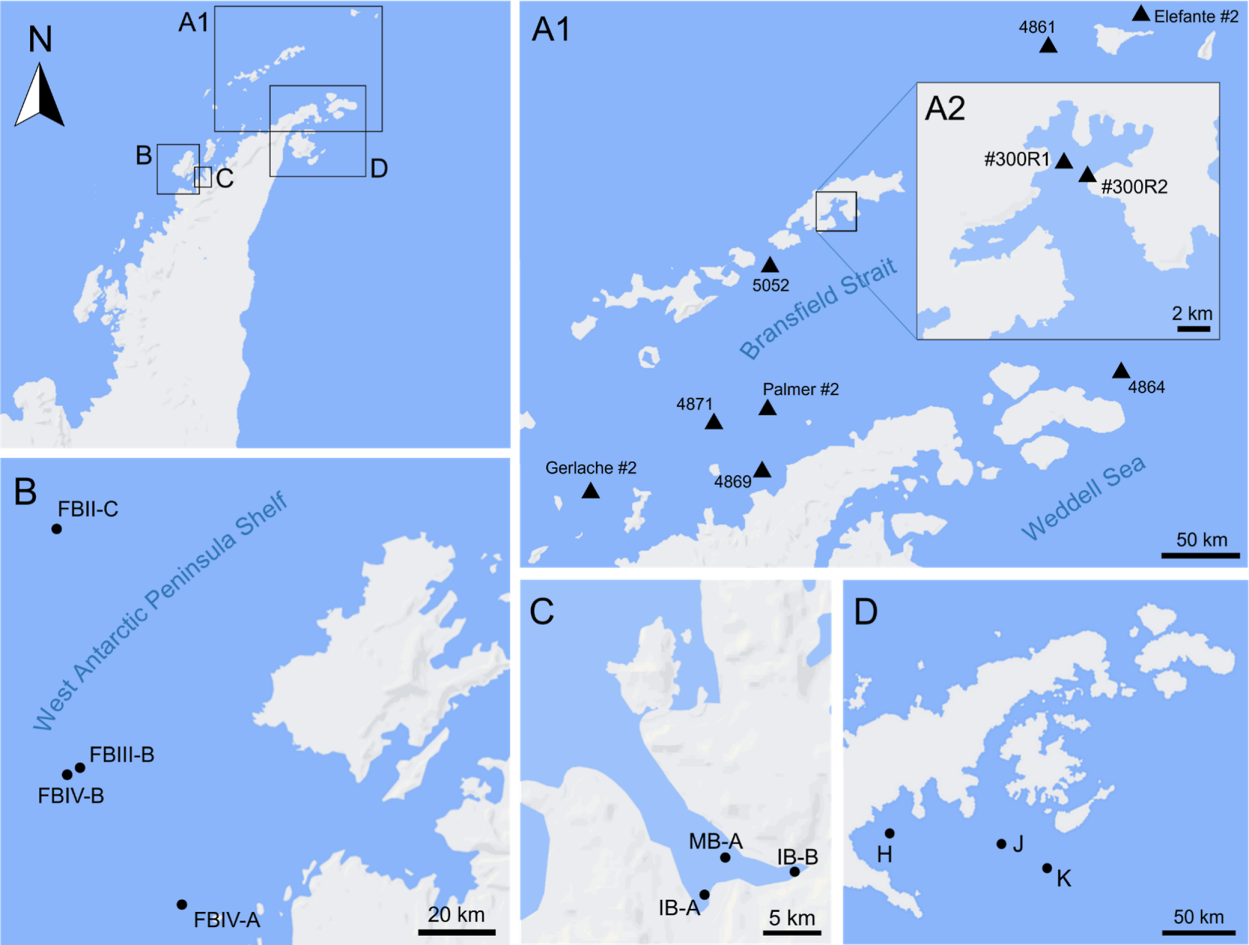
Study area depicting three decades of research efforts within the Brazilian Antarctic Program and the United States Antarctic Program, with sampling locations for deep-sea benthic invertebrates collected from the continental shelf surrounding the Antarctic Peninsula. A1. South Shetland Islands and northern Antarctic Peninsula. A2. Admiralty Bay, King George Island. B. West of Anvers Island, western Antarctic Peninsula. C. Andvord Bay, western Antarctic Peninsula. D. Tip of the Antarctic Peninsula and area of the former Larsen A Ice Shelf. Triangles indicate Universidade de São Paulo (ColBIO) sampling stations, circles indicate University of Hawai’i at Ma̅noa stations. More details regarding sampling stations can be found in Table S1.

Organisms were obtained during eight expeditions carried out within the Brazilian Antarctic Program (PROANTAR) and the United States Antarctic Program (USAP) in 1986, 1987, 2000, 2009, 2012, 2014, 2015, and 2016 (Table S1). Cruises covered the Bransfield Strait and the vicinity of Elephant and King George islands; west and southwest of Anvers Island and Andvord Bay; and the former Larsen A Ice Shelf (Figure 1). The Bransfield Strait comprises a body of water extending between the Antarctic Peninsula and the South Shetland Islands, and runs in a northeast-southwest direction. Elephant and King George are two of the South Shetland Islands, located north of the Antarctic Peninsula. Sampling in King George Island also took place within Admiralty Bay, south–southeast of the island. Anvers Island sits on the western shore of the Antarctic Peninsula, being the largest island in the Palmer Archipelago. Andvord Bay is situated east of Anvers Island, also in the West Antarctic Peninsula. Lastly, area A of the former Larsen Ice Shelf, located in the East Antarctic Peninsula, is a region which has been undergoing major ice shelf breakups since the mid-1800s.

In the 1980s, beam- (1.55 × 0.5 m, 5 mm mesh) and otter-trawls (14 m wide, 45 mm mesh) were used for benthic sampling. Starting with the cruises from 2000, either a smaller 0.6 × 0.4 m, 20 mm mesh beam trawl, or a 0.6 × 0.4 m, 20 mm mesh Agassiz trawl were used. Organisms were fixated immediately after collection. These were either stored in a 4% or 10% formalin solution, then transferred to 70% ethanol in glass jars once on land, or fixated directly in ethanol. Taxa were subsequently identified by specialists. In this work, only megafauna (>10 mm total length/diameter) samples were dissected and underwent analysis.

### 2.2 Biological Collections

Our research takes advantage of organisms collected as early as 1986 and catalogued in two archives: the Biological Collection “Prof. Edmundo F. Nonato” (ColBIO) at the Instituto Oceanográfico of the Universidade de São Paulo, obtained via the Brazilian Antarctic expeditions; and the University of Hawai’i at Ma̅noa marine sciences collection, sampled by Prof. Craig Smith through the United States expeditions. Organisms belonging to 15 species were analyzed (Table S1). These were selected according to three conditions: 1. collection from a depth greater than 200 m, which constitutes the standard definition of “deep sea”;^52^ 2. availability of multiple organisms per station to provide a baseline level of comparison within the same species; and 3. the largest possible variation in sampling locations and years. Feeding guilds were determined according to the dominant feeding mode following the literature for these species (Table 1). For a broad definition of the feeding modes reported here, refer to Table S2. When information was unavailable, feeding strategy was inferred based on literature records from either the same species found elsewhere, or evidence from congeneric species.

**Table 1:** Fibers in Deep-Sea Benthic Invertebrates Sampled from the Antarctic Continental Shelf and Deposited in the Universidade de São Paulo (ColBIO) and University of Hawai’i at Ma̅noa Collections^a^

species (n)	year	mass (g)	length (mm)	feeding mode	fibers (n)	fiber length (mm)	fiber color	fiber ind^–1^	fiber mass^–1^	ingestion (%)
*Echinopsolus koehleri* (6)	1986	1.12–3.08	23.18–31.26	suspension feeder^58,59^	8	0.36–5.91	blue (4), black (2), white (2)	1.33 ± 0.71	0.52 ± 0.24	50
Boreomysis sp. (10)	1986	0.65–1.00	52.37–66.92	suspension feeder, occasional predator^60^	3	1.69–2.33	blue (2), green	0.3 ± 0.15	1.04 ± 0.86	33
*Heterocucumis steineni* (5)	1986	2.65–9.14	30.78–59.40	suspension feeder^61^	6	0.57–4.08	blue (4), blue/white, orange	1.2 ± 0.2	0.24 ± 0.05	100
Notocrangon antarcticus (33)	1987, 2016	2.3–11.9	69.18–134.23	predator^62,63^	4	0.24–1.66	black (2), green (2)	0.12 ± 0.07	0.02 ± 0.01	0.09
*Harpovoluta charcoti* (10)	1987	3.94–12.55	32.46–50.59	scavenger^64^	2	0.79–1.31	black, light blue	0.2 ± 0.2	0.05 ± 0.05	10
*Protelpidia murrayi* (3)	2000	14–19.4	49.11–73.71	deposit feeder^65^	1	0.681	blue/white	0.33 ± 0.33	0.02 ± 0.02	33
*Scotoplanes globosa* (4)	2000	22.5–50.7	56.26–91.26	deposit feeder^65^	7	0.31–1.65	green (3), red (4)	1.75 ± 0.75	0.05 ± 0.02	75
*Molpadia violacea* (6)	2000, 2014	4.7–58.22	67.23–229.73	deposit feeder^67^	8	0.26–4.71	black (4), blue, green, red, white	1.33 ± 0.33	0.1 ± 0.07	83
*Ophionotus victoriae* (35)	2009, 2012, 2015	0.66–9.4	13.81–35.47	deposit feeder, scavenger, occasional predator^68−70^	21	0.25–4.04	blue (8), black (6), red (2), green (3), light blue, white	0.6 ± 0.18	0.24 ± 0.1	37
*Laetmonice producta* (6)	2009	0.26–37.24	20.54–173.12	predator, occasional scavenger/deposit feeder^71−73^	1	2.65	blue	0.1 ± 0.1	0.003 ± 0.003	10
*Amphioplus peregrinator* (17)	2014	0.81–2.22	10.79–18.45	suspension feeder, occasional deposit feeder^74,75^	17	0.17–5.06	blue (10), black (5), light blue, red	1 ± 0.33	0.7 ± 0.23	53
*Ophiosparte gigas* (10)	2012	6.7–19.4	31.46–42.22	predator, occasional scavenger^76,77^	4	0.74–3.97	blue (3), red	0.4 ± 0.16	0.02 ± 0.01	40
Nematocarcinus lanceopes (4)	2014	3.05–4.63	111.18–119.73	scavenger, occasional predator^78,79^	3	0.47–1.81	blue (2), green	0.75 ± 0.48	0.21 ± 0.13	50

a Fibers are shown in relation to invertebrate species, year of sampling, invertebrate mass and length ranges, feeding strategy, number of fibers, size of fibers, color of fibers, number of fibers per species (individual and wet weight; mean ± S.E.), and percentage of individuals which ingested at least one fiber.

**Table 2:** Microplastics in Deep-Sea Benthic Invertebrates Sampled from the Antarctic Continental Shelf and Deposited in the Universidade de São Paulo (ColBIO) and University of Hawai’i at Ma̅noa Collections^a^

Species	Date (dd/mm/yy)	Polymer	Fiber length (mm)	Fiber Color	Station
*Boreomysis* sp	02/02/1986	Polysulfone	2.13	Blue	4864
*Heterocucumis steineni*	08/02/1986	Polycarbonate + polyethylene terephthalate	0.57	Blue	4869
*Scotoplanes globosa*	16/06/2000	Polyamide	0.28–0.71	Red	FBIII-B
*Scotoplanes globosa*	??/10/2000	Polyisoprene	0.37–0.41	Red	FBIV-B
*Molpadia violacea*	29/10/2000	Polyamide + polyester	0.61	Blue	FBIV-A
*Ophionotus victoriae*	11/12/2009	Polycarbonate + polyethylene terephthalate	0.61	Blue	#300R1
*Ophiosparte gigas*	22/03/2012	Polyamide	0.74	Red	K

a Species, date of collection, polymer type, size of fiber, color of fiber and station identifiers are presented for sampled megafauna.

### 2.3 Laboratory Work

#### 2.3.1 Quality Assurance/Quality Control (QA/QC)

The samples processed here were not originally obtained with the intent of MD assessment, nor were the rigorous contamination measures implemented today enforced at the time. So, given the possibility of contamination during capture, handling and storage of historical samples, only intact specimens were considered in this study, i.e. all surveyed specimens had fully preserved outer body walls and wholly intact guts. Additionally, all investigated specimens were fixated on-site immediately after sampling took place, thus reducing the possibility of ingestion/egestion of fibers after collection,^53^ and providing us with a snapshot of the environmental conditions at the time of sampling. Prior to dissection, specimens were thoroughly rinsed with ultrapure water (resistivity of 18.2 MΩ·cm, Thermo Scientific Barnstead Easypure II) and inspected under a stereomicroscope (Nikon SMZ-1B, 30× total magnification). Intact, whole guts were then placed in a clean Petri dish and immersed in ultrapure water. Before dissection of the gut content, each sample was checked one more time for particles floating around the isolated organ. Any particles found were kept for further analysis (see next section).

To minimize present-day background contamination, samples were handled in a clean, enclosed and isolated laboratory with limited access to personnel. During analysis, the air conditioner was turned off, and doors and windows remained shut. All surfaces on the workbench were cleaned with filtered (100 μm) 70% ethanol on nonshredding paper three times and allowed to fully dry prior to analysis. Natural fiber clothes were worn under a clean cotton laboratory coat which never left the laboratory. Primarily metal and glass instruments were used, with as little plastic apparatus as possible. All instruments were thoroughly rinsed with ultrapure water prior to being used, and samples remained covered by a clean glass cover whenever necessary to limit air exposure.

Two additional procedures were undertaken to check for background contamination. Tape lift screenings (TLS) consist of using adhesive tape to assess workbench cleanliness: following alcohol cleanup, three 5 × 4 cm (60 cm^2^ total surface) pieces of clear adhesive tape were randomly placed face down on the surface of the workbench and then carefully removed and adhered to a clean Petri dish. Pieces of adhesive tape were then examined under a stereomicroscope. Any particles found were kept for further analysis. Procedural blanks consisted of a fiberglass filter (Whatman, 47 mm in diameter, 0.7 μm pore size) dampened with ultrapure water (dampened filter paper, DFP) and placed in a Petri dish before each work session to check for airborne contamination. Filters were confirmed to be clean prior to and after being dampened. Likewise, filters were inspected under a stereomicroscope and any particles found were kept for further analysis. Both protocols were based on Woodall et al. (2015).^54^ Background contamination was checked 45 times during this study, with an average of 3.75 processed samples per contamination control procedure (TLS + DFP). Finally, following Tirelli et al. (2022)^14^ and Bergami et al. (2023),^27^ procedural contamination was corrected by excluding any MD which shared similar characteristics (i.e. shape, color or polymer) to particles found in the background controls and blanks, from every corresponding batch of samples processed on that day.

#### 2.3.2 Isolation of Anthropogenic Microdebris

Specimens were precautiously weighed while still dampened using a precision scale (Shimadzu UX620H), as the measured mass of preserved specimens may be highly variable due to ethanol evaporation (minimized here by room temperature being kept constant), and then photographed. Photographs were later used for measurements of total length (most organisms) or central disk length (ophiuroids) using ImageJ 1.53n (National Institutes of Health). Whole gastrointestinal tracts – or stomach in the case of ophiuroids – were carefully removed with small incisions so not to cause excessive damage to the preserved specimens, and then analyzed under a stereomicroscope.

All particles found within the gut content as well as with TLS and DFP protocols were photographed and then measured using ImageJ. The identification key presented in Lusher et al. (2020)^55^ was followed to aid in the classification of particles as possibly anthropogenic, and all particles were classified by shape, size, color and elasticity. Following visual characterization, particles were wrapped in fiberglass filters held shut using a metal paperclip and immersed in 15% hydrogen peroxide (H_2_O_2_) (Êxodo) for 8 days to remove excess organic matter,^56^ before drying off in an oven (60 °C, Nova Ética 400 ND). Finally, particles were transferred to an Anodisc filter membrane (Whatman, 25 mm, 0.02 μm) and enclosed in a Petri dish.

#### 2.3.3 Polymer Identification

The Raman spectra were obtained using either an alpha300 R (WITec) microscope or an inVia (Renishaw) microscope with varying laser wavelengths and objective lenses (633 nm/50 x; 785 nm/100 x, respectively). Regarding the μFTIR spectra, these were obtained using either a HYPERION (Bruker Optics) microscope in the 1250–4000 cm^–1^ range, coupled with a 400–4000 cm^–1^ KBr beamsplitter and a DLaTGS high sensitivity detector (registered with either 128 or 256 accumulations and a resolution of 4 cm^–1^), or obtained via ATR mode with germanium as the reflection element, in the 700–4000 cm^–1^ range (with 64 accumulations).

### 2.4 Statistical Analysis

We tested whether specimen size and feeding mode could predict the number of ingested particles. We had the option to choose mass or length data to describe size, but decided to only include length in the analysis given the two variables are correlated, and to avoid problems related to possible variation in the mass of preserved specimens (due to ethanol evaporation). Normality was tested using the Shapiro-Wilk test and a log transformation was employed to decrease bias as length was not normally distributed. We conducted Generalized Linear Models (GLMs) to test the response variable (number of ingested particles) as a function of feeding mode and length, using the Poisson distribution. Single- and multipredictor models were used by 1. considering only length or feeding mode as the predictor variable; and 2. taking into account length plus feeding mode as predictor variables, respectively. Outliers in the number of particles were determined by using Grubbs’ test, and models were conducted with and without the outliers. We then tested pairwise differences regarding feeding modes using a post hoc Tukey’s test. Lastly, we used a *t*-test to investigate the difference between the mean size of fibers ingested by the two most representative groups, echinoderms and crustaceans. All statistics were carried out in R 4.4.1^57^.

## 3 Results

### 3.1 QA/QC

Particles found via TLS and DFP were 100% composed of fibers. The total number of background fibers was 84, or 1.87 ± 0.24 (mean ± S.E.) per day of sampling. Following immersion in H_2_O_2_, most background fibers either disintegrated or became susceptible to crushing under pressure and were not considered synthetic. Ten background fibers retained their original characteristics and underwent polymeric characterization. These were classified as polyacrylonitrile, polyamide and polyester blend, polyamide and polypropylene blend, polycarbonate, polyester, polyethylene (n = 2 fibers), polysulfone, polypropylene, and polyisoprene (synthetic rubber).

### 3.2 Microdebris in Deep-Sea Invertebrates

A total of 148 particles were recovered from 74 of the 169 sampled organisms. Fibers were the majority of MD (n = 146), followed by fragments (n = 2). Similar to particles found in the QA/QC, immersion in H_2_O_2_ resulted in the partial disintegration of some of the ingested particles, which were then discarded. Finally, ingested particles which could not be identified (see below) were compared against the blanks and also discarded if similar in color. We thus removed 61 fibers and the 2 fragments from our samples. Given this conservative approach, 85 fibers were found in 53/169 organisms, with an overall ingestion rate of 31.36% and an average ingestion of 0.50 ± 0.07 fiber ind^–1^. Fiber morphology and color are shown in Table 1 in accordance with the visual inspection. An additional visualization of how fiber ingestion relates to fiber color is provided in Figures S1 and S2. Fibers in megafauna were mostly blue (41%, n = 35), followed by black (24%, n = 20), green (13%, n = 11), red (11%, n = 9), white (5%, n = 4), light blue (4%, n = 3), blue/white (2%, n = 2) and orange (1%, n = 1).

**Figure 2:**
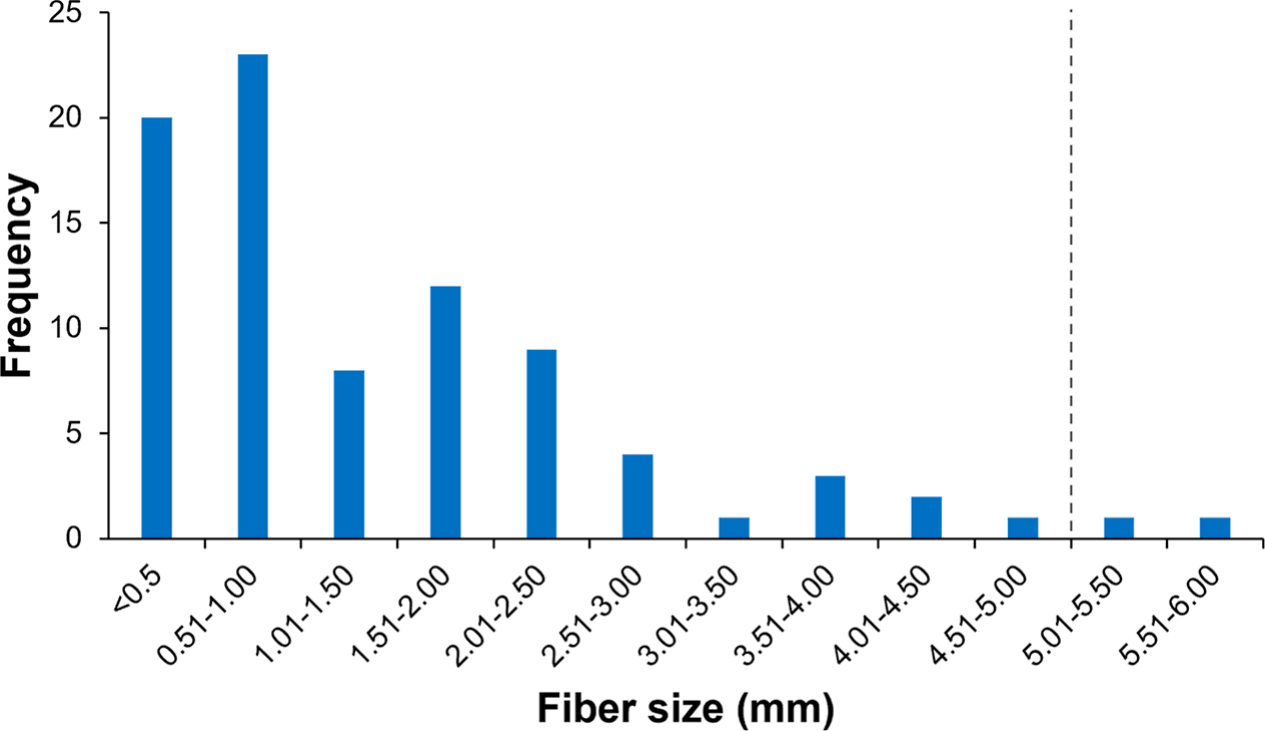
Size distribution of fibers (length, in mm) ingested by deep-sea benthic invertebrates sampled from the Antarctic continental shelf and deposited in the Universidade de São Paulo (ColBIO) and University of Hawai’i at Ma̅noa collections. The dotted line delimitates the upper limit for classifying fibers as microdebris (<5 mm).

Fibers ranged in size from 0.17 to 5.91 mm (mean length of 1.45 ± 0.13 mm), and most fibers were placed within the “micro” range, i.e. smaller than five mm (97.64%, n = 83) (Figure 2). Length was used as the standard measurement for most fibers. Only two fibers exceeded the size threshold, but these were also grouped as MD by using diameter measurements instead (for more details on microparticle measurement, see Schnepf et al., 2023^80^). Even though diameter is not usually considered the most appropriate measurement for the fibrous shape – due to a fiber’s elongated nature and small diameter not accurately representing its true size −, we chose this approach for the sake of simplicity. Therefore, by considering their maximum Feret’s diameter, i.e. length of the shortest line joining two points of an object, these two above-mentioned fibers were considered to be in the MD size range.

Fifty-seven of the ingested fibers were subjected to FTIR/Raman spectroscopy. Six polymers were identified, and all of them were classified as plastics (*n* = 7) (Table 2 and Figure S3). The remaining fibers were either unidentified or yielded dye spectra. Polymers were identified as polyamide (*n* = 2, 3.51%), polyamide and polyester blend (*n* = 1, 1.75%), polycarbonate and polyethylene terephthalate (PET) blend (*n* = 2, 3.51%), polysulfone (*n* = 1, 1.75%), and polyisoprene (synthetic rubber) (*n* = 1, 1.75%). MPs originating from the background controls or the blanks did not match ingested MP occurrences in the examined specimens within each sampling day, with the exception of a polyacrylonitrile fiber (found in an *Amphioplus peregrinator* individual from station Gerlache #2). Thus, this single MP occurrence was not considered in our work.

The organisms studied here are generally considered predators (39%), deposit feeders (28.4%), suspension feeders (22.49%) and scavengers (10%). Deposit feeders ingested 43.53% of fibers (n = 37 fibers), followed by suspension feeders (40%, n = 34), predators (10.59%, n = 9) and scavengers (5.88%, n = 5). Brittle stars and sea cucumbers concentrated 83% of ingested fibers (n = 72), having ingested 42 and 30 fibers respectively. All *Heterocucumis steineni* sea cucumber individuals (n = 5) had at least one fiber in their gut. Only one individual was found to have ingested fibers in *Harpovoluta charcoti* gastropods (n = 10, 10% of individuals) and *Laetmonice producta* polychaetes (n = 10, 10%). Conversely, *Eusirus perdentatus* amphipods (n = 3) and *Chorismus antarcticus* shrimp (n = 13) had no fibers within their gut content. Deposit- and suspension-feeding accounted for 83.53% (71/85) of fibers found in this study.

The oldest confirmed MP record was obtained from a *Boreomysis* sp. mysid sampled on the second of February, 1986, which ingested a blue, 2.13 mm long polysulfone fiber. Average fiber ingestion for all invertebrate species was 0.50 ± 0.07 fiber ind^–1^. Out of the four feeding modes analyzed, deposit- and suspension-feeding yielded the highest values for number of ingested fibers, in descending order: in *Scotoplanes globosa* (1.75 ± 0.75 fiber ind^–1^), *Molpadia violacea* (1.33 ± 0.33 fiber ind^–1^), *Echinopsolus koehleri* (1.33 ± 0.71 fiber ind^–1^), *H*. *steineni* (1.2 ± 0.2 fiber ind^–1^), *A. peregrinator* (1.00 ± 0.33 fiber ind^–1^), and *Ophionotus victoriae* (0.6 ± 0.18 fiber ind^–1^). The lowest values were observed, in ascending order: in the predacious polychaete *L. producta* (0.10 ± 0.10 fiber ind^–1^), the predacious shrimp *Notocrangon antarcticus* (0.12 ± 0.07 fiber ind^–1^), the scavenging gastropod *H. charcoti* (0.2 ± 0.2 fiber ind^–1^), the suspension-feeding mysid *Boreomysis* sp. (0.3 ± 0.11 fiber ind^–1^), and the predacious brittle star *Ophiosparte gigas* (0.4 ± 0.16 fiber ind^–1^) (Table 1).

**Figure 3:**
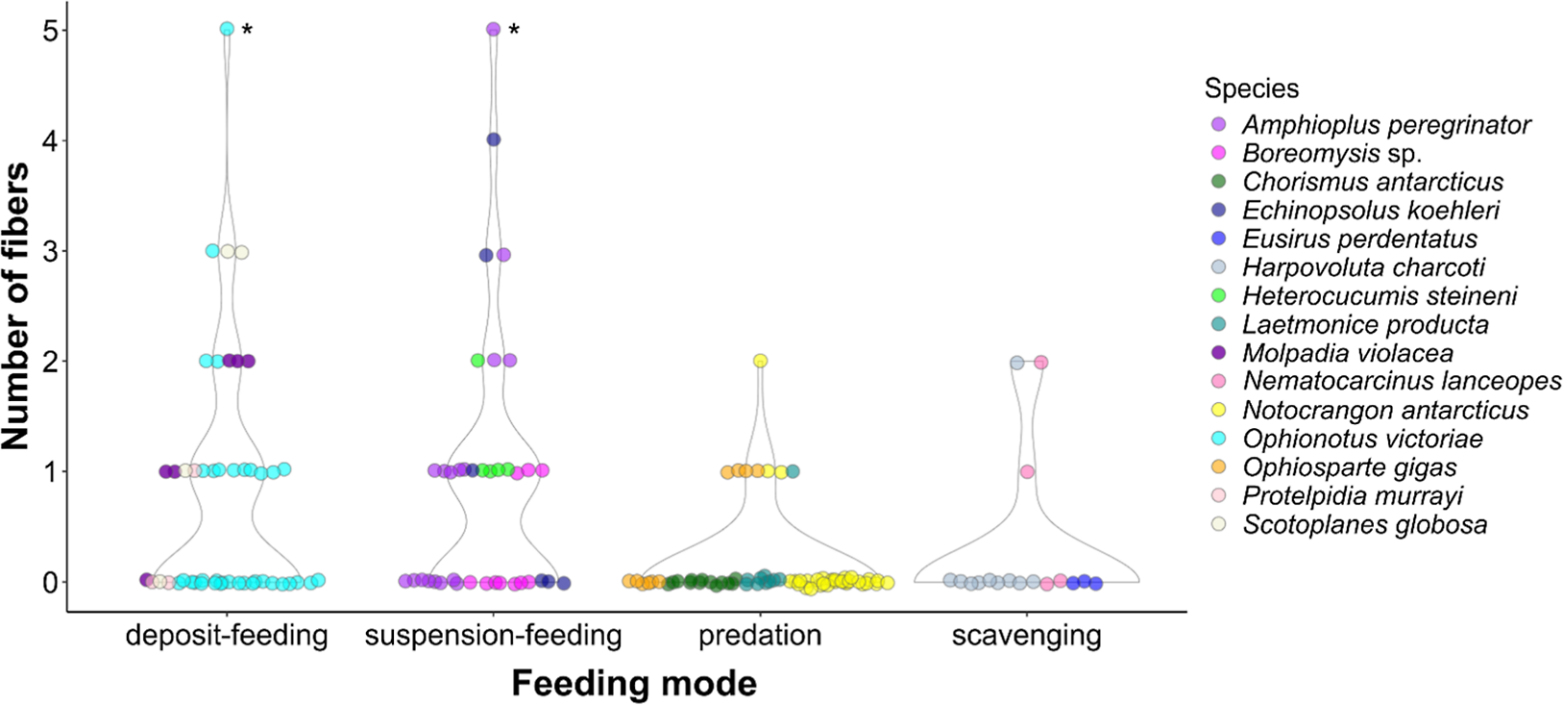
Number of fibers across the four feeding modes commonly associated with deep-sea benthic invertebrates sampled from the Antarctic continental shelf and deposited in the Universidade de São Paulo (ColBIO) and University of Hawai’i at Ma̅noa collections. Each circle represents one individual and the number of fibers found in its gut content. Species are indicated by color, and asterisks indicate the outliers.

The association between the number of ingested particles and length or feeding mode resulting from the single-predictor models was similar (Table S3), with and without the outliers. We detected two outliers in the number of particles (G = 4.82, U = 0.86, *p* < 0.05), in the specimens which ingested five particles. The model considering only length showed a negative association with the number of ingested fibers and all individuals included (F = 6.742, *p* = 0.008); the number of ingested particles thus decreased with organism size. Similarly, the single-predictor model for organism size without the outliers also showed a negative significant difference toward particle ingestion (F = 3.554, *p* = 0.027). The single-predictor models which considered feeding modes showed that feeding strategy was associated with the number of ingested fibers both when outliers were included (F = 8.157, *p* < 0.001) and not included (F = 8.011, *p* < 0.001). In the models which included the outliers, a significant difference occurred between predation and both deposit- and suspension-feeding (Tukey’s post hoc test: *p* < 0.05; Table S4). The same differences were found in the model without the outliers (Tukey’s test: *p* < 0.05). The multipredictor models followed a similar pattern, in which the number of ingested fibers was associated with feeding mode, and also associated with organism size for the models with the outliers (F_Size_ = 7.315, *p* = 0.007 and F_F. mode_ = 5.988, *p* < 0.001) and without the outliers (F_Size_ = 3.981, *p* = 0.048 and F_F. mode_ = 7.575, *p* < 0.001). Figure 3 depicts the variation in the number of ingested fibers across all feeding modes surveyed in our study, with each species represented by a unique color. Finally, there was no significant difference between the mean size of fibers ingested by echinoderms and crustaceans, the two most representative groups (t = −1.03, df = 15.779, *p* = 0.31, CI = [−0.95, 0.32]).

## 4 Discussion

We confirm the presence of anthropogenic microdebris in the gut content of deep-sea invertebrates collected between 1986 and 2016 along the shelf of the Antarctic Peninsula and available in the biological collections at the Universidade de São Paulo and the University of Hawai’i at Ma̅noa. The work of curators and researchers in providing precious environmental insights decades after sampling and the importance of biological archives^81^ are evidenced by our findings, which also show the oldest on-site record of a microplastic in Antarctica. Our findings indicate a prevalence of fibers in the benthic community of the Southern Ocean and offer more data on the widespread impact of human activities even in seemingly pristine regions. The largest organisms sampled here ingested fewer fibers, and though significant this effect was small. This indicates that other factors may be influencing the ingestion of MD, such as feeding mode. Finally, this study highlights deposit and suspension feeders as the group of benthic organisms which ingested the largest quantity of fibers across the most common feeding strategies.

Information on invertebrate feeding ecology is critical to understanding the distribution of MD in the ocean,^82^ being more commonly available for commercial species and fish.^43,83,84^ Despite only representing a third of the species surveyed in our study, deposit-feeding was the feeding mode which concentrated the highest portion of fibers (37/85, 43.53%), a similar result found by Bour et al. (2018)^85^ in a Norwegian fjord and Rios-Fuster et al. (2022)^86^ in the western Mediterranean Sea. Contrary to Bour et al. (2018),^85^ however, we did not observe a comparably high percentage of fiber ingestion in (epi) benthic predators, which could be related to differences in food type and abundance, and availability of MD between that region and our study area. Echinoderms, represented by the sea cucumbers *E. koehleri*, *H. steineni*, *M. violacea*, *Protelpidia murrayi* and *S. globosa*, and the brittle stars *A. peregrinator*, *O. gigas* and *O. victoriae*, contained 84.7% of all fibers found in our study, further indicating the role of this phylum as a reservoir for historical anthropogenic debris.^51^

The sea cucumbers *S. globosa, M. violacea, E. koehleri* and *H. steineni* were the species with the highest means of ingested fibers. These findings come as no surprise given how holothurians are capable of concentrating MPs available in the sediment^87,88^ and may even selectively ingest MP.^89,90^ In this regard, echinoderms can be considered excellent indicators of the concentration of anthropogenic MD on the seafloor, and should be capable of providing valuable insights when coupled with established shallow-water indicators, such as bivalves;^53,91^ for instance, the alarming reports by González-Aravena (2024)^92^ present the highest values for MD in Antarctic benthos thus far, with 42.86 ± 25.36 fibers and fragments ind^–1^ in the clam *Laternula eliptica*. Nevertheless, studies on the ingestion of MD by Antarctic invertebrates are restricted to shallower depths and have typically presented ingestion values in the range reported in our study. Sfriso et al. (2020)^26^ reported a maximum value of 1.9 fragment ind^–1^ for the suspension-feeding bivalve *Thyasira debilis*, and an overall average ingestion of 1.0 fragment ind^–1^ across bivalves, gastropods, amphipods, polychaetes and actinarians. Similarly, Bergami et al. (2023)^27^ reported 0.3 ± 0.53 textile fiber ind^–1^ in the Antarctic whelk *Neobuccinum eatoni*.

Conversely, predacious species consistently showed the lowest values of fibers per individual, despite being more numerous than the deposit feeders in our samples. The highest ingestion percentage in this guild was observed in *O. gigas* (0.4 ± 0.16 fiber ind^–1^), which is the only opportunistic predacious echinoderm in our study. A likely reason could be its ventral-facing mouth, which is in constant intimate association with the seafloor; small amounts of contaminated sediment could then be ingested while this brittle star forages on other benthic organisms. These field observations offer some interesting insights, especially when compared to laboratorial settings, where predacious crustaceans have been shown to accumulate MP particles ingested by their prey.^93−95^ Regarding the lower levels of fibers in scavengers, our findings could be biased by the lower number of analyzed specimens for this guild.

Although there was no significant relationship, we detected a tendency for smaller fibers in the mysids and shrimp (≤2.33 mm), while larger ones were found in the sea cucumbers and brittle stars (≤5.91 mm) (Figure S4). Gut morphology in crustaceans, with the presence of a gastric mill and a complex filtering system of setae, could be an impediment for the passage of larger fibers from the stomach to the intestine in this group, as seen in laboratory^96,97^ and in field studies.^98^ Dawson et al. (2018)^97^ demonstrated how plastic particles in the “micro” size range can be further broken down to the “nano” range through mechanical digestion in the Antarctic krill, with severe implications for the species which prey upon these keystone organisms. While still in the “micro” range, a fifth of our fibers were smaller than 0.5 mm in length, which could also lead to entanglement in the swimming appendices and reduced motility of the zooplankton.^99^

The dominance of fibers in our samples is to be expected given the previously documented environmental prevalence of such items. Woodall et al. (2014)^11^ showed that fibers were up to 4 orders of magnitude more abundant in deep-sea sediments than in contaminated sea surface waters, and fibers are indeed generated by many aspects of everyday life.^100−102^ Since studies on Antarctic fibers usually focus on samples collected in proximity to scientific stations,^103^ those fibers are likely originating from laundry-released wastewater. Textile fibers have now been found in Antarctic sediment^15,104−107^ and water samples.^108−110^ Regarding MD in Antarctic marine invertebrates, fibers were also the most common shape found by Bergami et al. (2023),^27^ Wilkie Johnston et al. (2023),^24^ Zhu et al. (2023)^25^ and González-Aravena (2024),^92^ while Sfriso et al. (2020)^26^ reported mostly fragments.

Polyester and polyamide were the most prevalent polymers in our samples, both being the main constituents of garments worn by researchers during outdoor activities in Antarctica.^27^ Polyethylene terephthalate (PET), a thermoplastic in the polyester family, is the most common constituent of MP fibers according to a global survey on surface waters,^109^ and was likewise the principal material ingested by gastropods in Terra Nova Bay.^27^ Polyamide was also the most abundant material in floating fibers obtained around the Antarctic Peninsula,^111^ in zooplanktonic Antarctic krill and salps sampled around South Georgia^24^ and in the benthic invertebrate community of Terra Nova Bay.^26^ While PET and PA in the shape of fibers are widely used in textiles,^112^ polycarbonate and PET blends are in turn typically used in electrical and electronic applications due to their cold resistance properties.^113^ Similarly, the polysulfone in our oldest fiber is a thermoplastic which is widely used in plumbing, electrical applications and membrane filters.^114^ In this case, a textile origin for these materials is unlikely even though they were found as fibers in the present study. So, while laundry effluents from research stations and tourism/scientific vessels are indeed a major source for textile fibers in Antarctic samples,^23,108,115^ other local sources for nontextiles cannot be excluded, as well as atmospheric and ocean circulation.^17,18,105^

Due to high levels of endemism in the Southern Ocean, the Antarctic Polar Front (APF) has long been established as a barrier to the movement and migration of organisms as well as floating debris.^116^ A growing number of works, however, have brought this notion into dispute (Murphy et al. 2021^117^ and references therein), with evidence of MD crossing into Antarctic boundaries.^118^ Therefore, when postulating alternatives to the local origin of fibers, the possible biologically driven transport of MD between Antarctic and Subantarctic regions is also worthy of mention. Since the APF is essentially a surface feature,^119^ the deep-sea benthos does not seem to be as affected by it,^120−124^ at least in comparison to organisms with pelagic or planktonic larval stages. In this regard, mobile bottom-dwelling invertebrates like sea cucumbers and brittle stars, many of which can cover several kilometers a day either via actively swimming^125,126^ or tumbling/floating above the seafloor,^127^ could be playing a role as vectors of MD across oceanographic borders in Antarctica and elsewhere.

Studies reporting the ingestion of anthropogenic MD by organisms in the Arctic can provide valuable insights given the similarity between the Earth’s two polar regions. When compared to Southern Ocean food webs, organisms in the shallower continental shelves of the Arctic Ocean rely even further on the benthopelagic coupling caused by marine snow,^18,128^ which could result in an exacerbation of the effects of MD ingestion. The number of ingested fibers in our study (0.1–1.75 fiber ind^–1^) is similar to reports from the Bering and Chukchi Seas benthos by Fang e al. (2018),^129^ which found 0.04–1.67 fiber ind^–1^ in 11 species of invertebrates including sea stars, shrimp, crabs, gastropods and bivalves; in that study, however, a predacious organism, i.e. the star *Asteria rubens*, ingested most of the fibers. Similar values were also obtained in deep-sea suspension-feeding polychaetes (0.10–1.9 particle ind^–1^) from the Norwegian continental shelf^130^ and in the deposit-feeding sea star *Ctenodiscus crispatus* from the Chukchi Sea (0.1–1.4 particle ind^–1^).^131^ The concentration of ingested fibers per organism in our study is thus comparable to levels observed in the opposite polar region. This raises concerns considering the stark difference in human population density between these areas: over 4 million people live in the Arctic, while Antarctica has a maximum of 10,000 visiting researchers and support staff during summer and only 1000 in winter.^132^

Considering the highly conservative approach applied to our study, we must emphasize that the findings reported here may have led to an underestimation of MD items, since only a small number of fibers could be chemically characterized. It is also likely that the H_2_O_2_ pretreatment eliminated fibers that would have counted toward the total number. We thus recommend employing alternative pretreatments to avoid loss of materials. We also reiterate that our samples were not originally collected with the specific intent of analyzing MD, and that using intact organisms was paramount to providing robust evidence of historical anthropogenic impacts, given the possibility of past contamination.

As scientists who care deeply about Antarctica for its intrinsic value, we recommend that others dedicating their time to the study of MD in the region take part in cross-disciplinary groups such as the Plastics in the Southern Ocean Expert Group (PLASTICS-EG),^133^ the DOSI Pollution and Debris Working Group^134^ and the Scientists’ Coalition for an Effective Plastics Treaty.^135^ And as professionals who are fortunate enough to work in the field of polar research, we must humbly consider how our very own footprint impacts the Antarctic ecosystem. We thus call for continued efforts to monitor and mitigate fibers released through wastewater in vessels and research stations. However, we stress that while half of the stations in Antarctica have no adequate water treatment facilities,^115^ laundry effluents are not the only source of fibers to the region. Baseline studies on the ecology of the Antarctic benthos are also necessary to support future assessment of MD ingestion according to feeding mode and, ultimately, trophic level. Contrary to holothurians, ophiuroids display a wide range of feeding strategies, sometimes within the same species,^75,136^ and feeding modes may even differ across environments. Hence, the inclusion of organisms from other regions of the deep shelf, especially from East Antarctica, is of critical importance. Finally, studies seeking to evaluate temporal trends in MD occurrence by means of archives should consider minimizing the destruction of invaluable collection specimens,^137,138^ as we have done here.

Our study shows, to the best of our knowledge, the oldest on-site occurrence of a microplastic in Antarctica, a polysulfone fiber found in a mysid sampled off the deep Southern Ocean shelf in 1986. All subsequent expeditions, encompassing a 30-year period, also yielded organisms which had ingested fibers. Our data highlights the relevance of deposit and suspension feeders – especially echinoderms – as indicators of anthropogenic MD in the deep sea and showcase how these organisms can complement information from well-established shallow-water indicators. Nevertheless, given the accelerated construction of scientific research stations starting from the latter half of the twentieth century (so after the advent of plastic production), MP pollution in Antarctica is very likely to precede the 1980s. The findings reported here are then but a glimpse into the occurrence of MD in the waters and sediments of the world’s most remote continental shelf. In addition to assessing present-day contamination, future studies can benefit from using historical samples across different matrices – such as sediment and ice cores – along with biological archives. A worrying trend is that while publications using biological collections to help elucidate temporal tendencies have been on the rise within the last three decades, support for the maintenance of such collections is facing diminishing funding.^139^ Therefore, our data set, in addition to constituting a testimony to the broad scale of anthropogenic impacts on remote ecosystems, also demonstrates the value of archiving and documenting biological samples for posterity.

## Supporting Information Available

•Species list and field sampling details (PDF); definition of feeding modes (PDF); number of fibers by color across different groups (PDF); representation of Pearson residuals (PDF); stereomicroscopic picture of fibers identified as microplastics (PDF); GLM values (PDF); post hoc Tukey’s test values (PDF); fiber size in crustaceans and echinoderms (PDF) The Supporting Information is available free of charge at https://pubs.acs.org/doi/10.1021/acs.est.4c09487.
