# Supplementary material for: Bottom-Feeders Eat Their Fiber: Ingestion of Anthropogenic Microdebris by Antarctic Deep-Sea Invertebrates Depends on Feeding Ecology
Source: Environ Sci Technol. 2024 Nov 21;58(50):22355–67. doi: 10.1021/acs.est.4c09487 (PMC11656696; doi:10.1021/acs.est.4c09487)
Supplement: Supplementary file 1 — es4c09487_si_001.pdf [file es4c09487_si_001.pdf]

Supporting information for:

Bottom-feeders eat their fiber: ingestion of anthropogenic microdebris by Antarctic deep-sea invertebrates depends on feeding ecology

Gabriel Stefanelli-Silva[1*], Pâmela Friedemann[2], Beatriz Rocha de Moraes[3], Romulo Augusto Ando[3], Lúcia de Siqueira Campos[4], Mônica Angélica Varella Petti[1], Craig R. Smith[5] & Paulo Yukio Gomes Sumida[1]

1. Departamento de Oceanografia Biológica, Instituto Oceanográfico da Universidade de São Paulo (IO-USP), São Paulo, 05508-120, Brazil
2. Departamento de Ecologia, Instituto de Biociências da Universidade de São Paulo (IB-USP), São Paulo, 05508-090, Brazil
3. Departamento de Química Fundamental, Instituto de Química da Universidade de São Paulo (IQ-USP), São Paulo, 05509-900, Brazil
4. Departamento de Zoologia, Instituto de Biologia da Universidade Federal do Rio de Janeiro (IB-UFRJ), Rio de Janeiro, 2141-902, Brazil
5. Department of Oceanography, University of Hawai'i at Mānoa, Honolulu, Hawai'i, United States
*Corresponding author: gabrielstefanelli@hotmail.com

Summary of contents
Number of pages: 11
Number of figures: 4
Number of tables: 4

Table S1. Range of deep-sea benthic invertebrates sampled from the Antarctic continental shelf and deposited in the Universidade de São Paulo (ColBIO) and University of Hawai'i at Mānoa collections. Station identifiers, coordinates, number of dissected organisms in each station, date of collection, and trawling depth are presented for sampled megafauna. Respective programs, projects and vessels are also presented. PROANTAR – Programa Antártico Brasileiro; USAP – United States Antarctic Program; Bion Fauna Bent Ant – Bionomia da Fauna Bentônica Antártica; FOODBANCS – Food for Benthos on the Antarctic Continental Shelf; MABIREH – Antarctic Marine Life: Biodiversity in Relation to Environmental Heterogeneity; LARISSA – Larsen Ice Shelf System, Antarctica; SOBE – Sistemas de Observação Bentônicos no Oceano Austral; FjordEco – Fjord Ecosystem Structure and Function on the West Antarctic Peninsula.

Station	Coordinates	Species (n)	Date (dd/mm/yy)	Depth (m)	Program	Project	Vessel
4861	61°08' S; 55°52' W	*Echinopsolus koehleri* (Vaney, 1914), Holothuroidea (6)	01/02/86	362	PROANTAR	Bion Fauna Bent Ant	Prof. W. Besnard
4864	63°01' S; 54°49' W	*Boreomysis* sp. G.O. Sars, 1869, Malacostraca (10)	02/02/86	275	PROANTAR	Bion Fauna Bent Ant	Prof. W. Besnard
4869	63°33' S; 59°15' W	*Heterocucumis steineni* (Ludwig, 1898), Holothuroidea (5)	08/02/86	240	PROANTAR	Bion Fauna Bent Ant	Prof. W. Besnard
4871	63°16' S; 59°55' W	*Chorismus antarcticus* (Pfeffer, 1887), Malacostraca (6)	08/02/86	264	PROANTAR	Bion Fauna Bent Ant	Prof. W. Besnard
5052	62°26' S; 59°16' W	*Notocrangon antarcticus* (Pfeffer, 1887), Malacostraca (13)	24/02/87	212	PROANTAR	Bion Fauna Bent Ant	Prof. W. Besnard
5052	62°26' S; 59°16' W	*Chorismus antarcticus* (Pfeffer, 1887), Malacostraca (2)	24/02/87	212	PROANTAR	Bion Fauna Bent Ant	Prof. W. Besnard
5052	62°26' S; 59°16' W	*Eusirus perdentatus* Chevreux, 1912, Malacostraca (3)	24/02/87	212	PROANTAR	Bion Fauna Bent Ant	Prof. W. Besnard
5052	62°26' S; 59°16' W	*Laetmonice producta* Grube, 1877, Polychaeta (4)	24/02/87	212	PROANTAR	Bion Fauna Bent Ant	Prof. W. Besnard
5052	62°26' S; 59°16' W	*Harpovoluta charcoti* (E. Lamy, 1910), Gastropoda (10)	24/02/87	212	PROANTAR	Bion Fauna Bent Ant	Prof. W. Besnard
FBII-C	64°13' S; 65°25' W	*Protelpidia murrayi* (Théel, 1879), Holothuroidea (3)	10/03/00	550-569	USAP	FOODBANCS	Laurence M. Gould
FBIII-B	64°48' S; 65°17' W	*Scotoplanes globosa* (Théel, 1879), Holothuroidea (2)	16/06/00	609-628	USAP	FOODBANCS	Nathaniel B. Palmer
FBIV-B	64°49' S; 65°21' W	*Scotoplanes globosa* (Théel, 1879), Holothuroidea (2)	??/10/00	602-700	USAP	FOODBANCS	Laurence M. Gould
FBIV-A	65°07' S; 64°42' W	*Molpadia violacea* Studer, 1876, Holothuroidea (3)	29/10/00	444-658	USAP	FOODBANCS	Laurence M. Gould
#300R1	62°06' S; 58°26' W	*Ophionotus victoriae* Bell, 1902, Ophiuroidea (10)	11/12/09	338	PROANTAR	MABIREH	Ary Rongel
#300R2	61°08' S; 55°52' W	*Laetmonice producta* Grube, 1877, Polychaeta (6)	11/12/09	316	PROANTAR	MABIREH	Ary Rongel
K	64°58' S; 57°46' W	*Ophiosparte gigas* Koehler, 1922, Ophiuroidea (5)	22/03/12	422-445	USAP	LARISSA	Nathaniel B. Palmer
H	64°48' S; 60°12' W	*Ophionotus victoriae* Bell, 1902, Ophiuroidea (5)	31/03/12	510-592	USAP	LARISSA	Nathaniel B. Palmer
J	64°39' S; 58°20' W	*Ophiosparte gigas* Koehler, 1922, Ophiuroidea (5)	07/04/12	542-543	USAP	LARISSA	Nathaniel B. Palmer
Palmer #2	63°12' S; 59°17' W	*Molpadia violacea* Studer, 1876, Holothuroidea (3)	14/01/14	805	PROANTAR	SOBE	Almirante Maximiano
Elefante #2	60°55' S; 54°52' W	*Amphioplus peregrinator* (Koehler, 1912), Ophiuroidea (7)	17/01/14	734	PROANTAR	SOBE	Almirante Maximiano
Elefante #2	60°55' S; 54°52' W	*Nematocarcinus lanceopes* Spence Bate, 1888, Malacostraca (4)	17/01/14	734	PROANTAR	SOBE	Almirante Maximiano
Gerlache #2	63°41' S; 61°22' W	*Amphioplus peregrinator* (Koehler, 1912), Ophiuroidea (10)	20/01/14	749	PROANTAR	SOBE	Almirante Maximiano
IB-B	64°52' S; 62°26' W	*Ophionotus victoriae* Bell, 1902, Ophiuroidea (10)	06/12/15	557	USAP	FjordEco	Laurence M. Gould
IB-A	64°53' S; 62°34' W	*Ophionotus victoriae* Bell, 1902, Ophiuroidea (10)	16/12/15	534	USAP	FjordEco	Laurence M. Gould
MB-A	64°51' S; 62°34' W	*Notocrangon antarcticus* (Pfeffer, 1887), Malacostraca (10)	07/04/16	530	USAP	FjordEco	Nathaniel B. Palmer
MB-A	64°51' S; 62°34' W	*Chorismus antarcticus* (Pfeffer, 1887), Malacostraca (5)	07/04/16	530	USAP	FjordEco	Nathaniel B. Palmer
MB-A	64°50' S; 62°35' W	*Notocrangon antarcticus* (Pfeffer, 1887), Malacostraca (10)	24/04/16	433-450	USAP	FjordEco	Nathaniel B. Palmer

Table S2. General description for the feeding modes most commonly associated with the deep-sea benthic invertebrates sampled from the Antarctic continental shelf and deposited in the Universidade de São Paulo (ColBIO) and University of Hawai'i at Mānoa collections.

Feeding mode	Description
Predation	Predators attack and consume prey which is on the seafloor, or use their appendices to capture free-swimming prey
Scavenging	Scavengers consume carrion; scavengers may also use their appendices to forage on microalgae which has sunk to the bottom
Deposit-feeding	Deposit feeders consume sediment while grazing along the seafloor surface, capturing sediment particles with their appendices, or burrowing into soft sediments, thus ingesting living and nonliving organic matter
Suspension-feeding	Suspension feeders capture and consume food particles from the water column. Most suspension feeders employ an "aerosol" mechanism, i.e. by capturing particles smaller than the spacing between their feeding structures

Fibers in relation to color are represented in Figure S1. We used a chi-squared test to check for the association between fiber color and 1. all surveyed species; 2. holothurians and ophiuroids only; and 3. holothurians according to feeding mode (suspension- and deposit-feeding). The association between fiber color and all thirteen species was significant (Chi-squared = 143.03, df = 84, $p < 0.001$), with certain species showing different levels of contribution toward the association (Figure S2). For example, *H. steineni* and *P. murrayi* showed a positive association with fibers categorized as blue/white, since they were the only ones which ingested fibers of this color. *Heterocucumis steineni* also had a positive association with orange fibers, because it was likewise the only species which ingested fibers of this color. There was no significant association between the two echinoderm groups and fiber color (Chi-squared = 10.825, df = 7, $p = 0.14$). Meanwhile, there was a significant association between holothurian feeding mode and fiber color (Chi-squared = 16.384, df = 6, $p = 0.011$), with the two feeding modes presenting significant differences in the color of ingested fibers. Suspension-feeding was positively associated with blue, orange and white fibers. Deposit-feeding feeding was positively associated with green and red fibers.

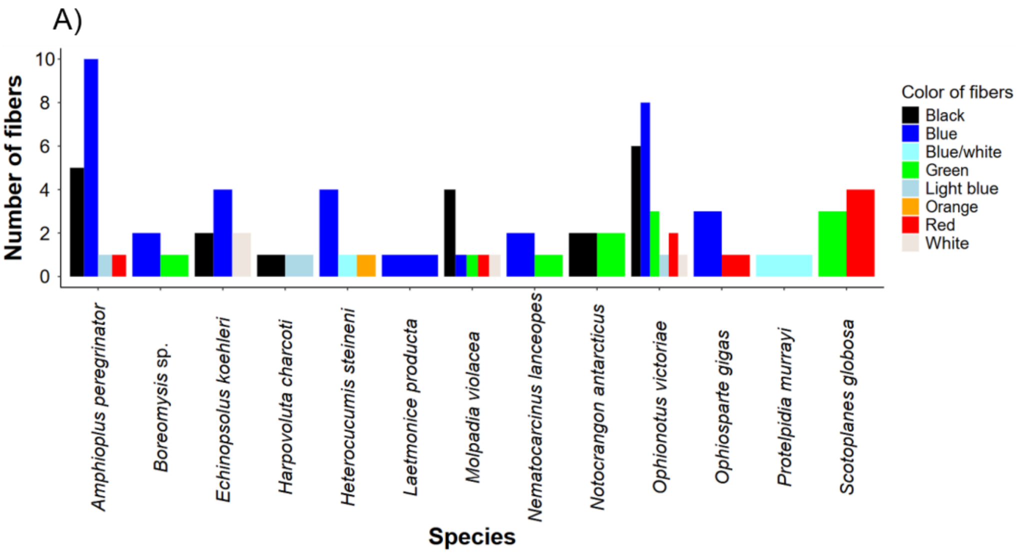

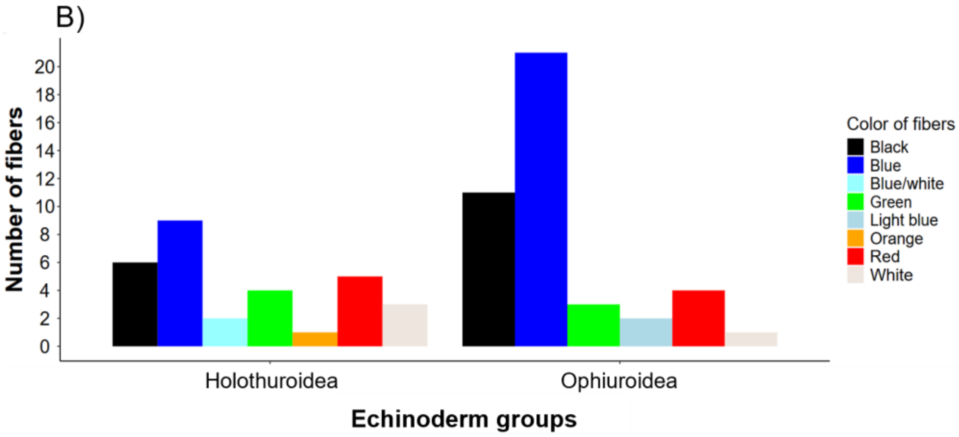

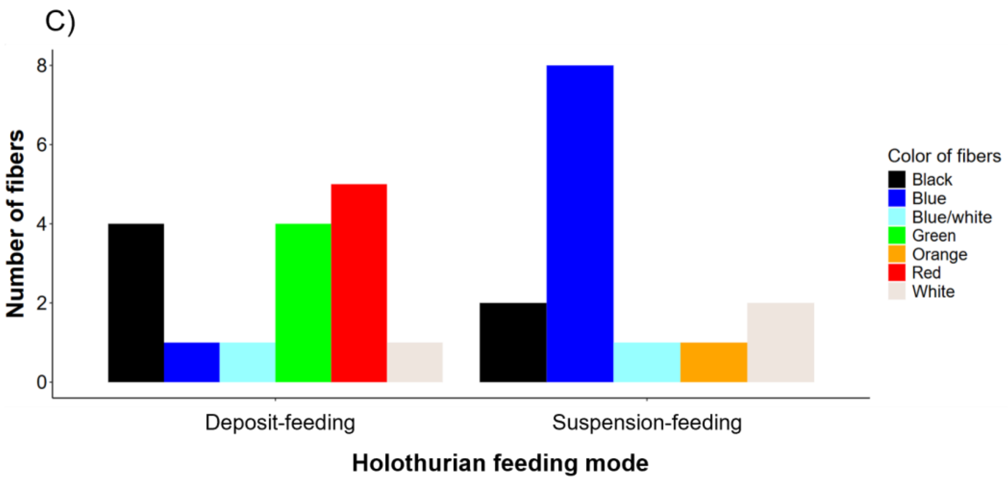

Figure S1. Number of fibers per color across all deep-sea benthic invertebrates sampled from the Antarctic continental shelf and deposited in the Universidade de São Paulo (ColBIO) and University of Hawai'i at Mānoa collections (A); in holothurians and ophiuroids only (B); and in relation to holothurian feeding mode (C).

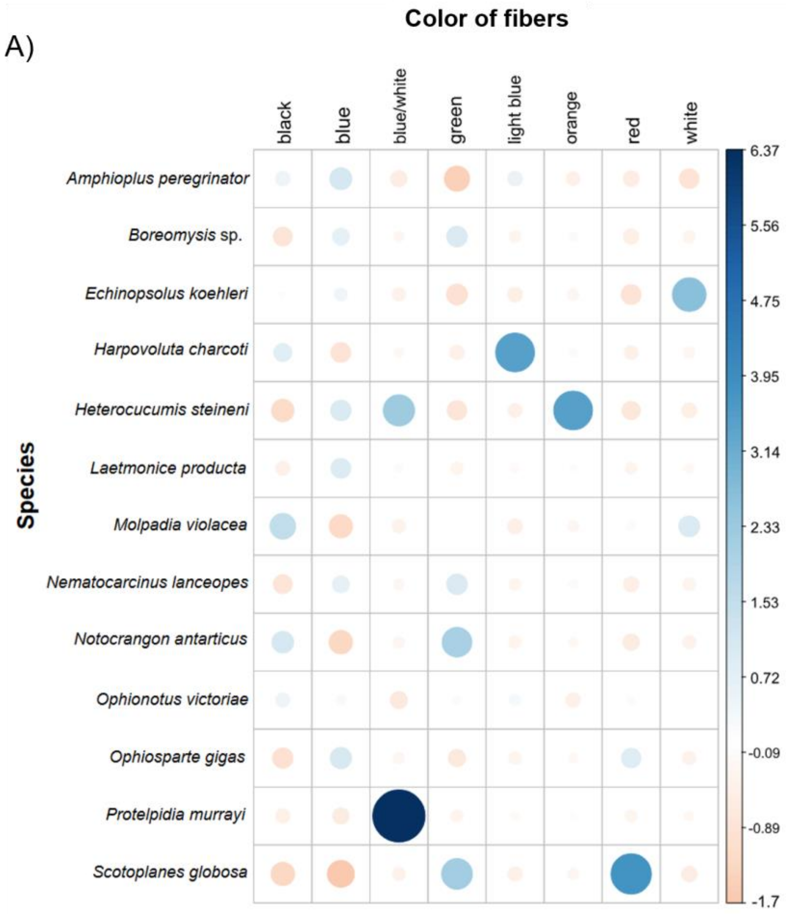

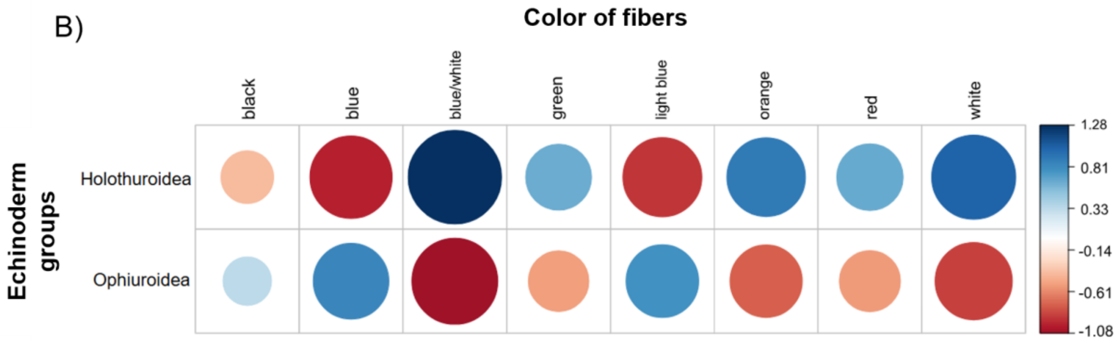

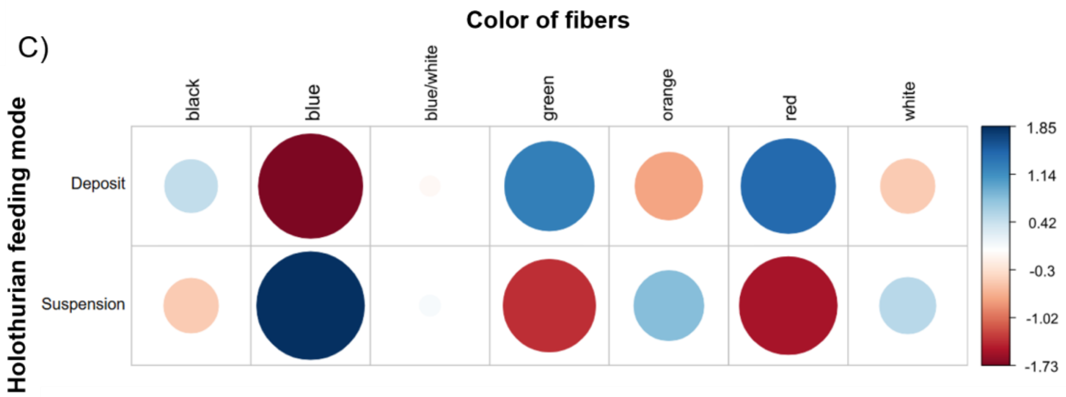

Figure S2. Pearson residuals extracted from the Chi-squared test to evaluate the association between fiber color and all surveyed species (A); fiber color and holothurians and ophiuroids only (B); and fiber color and holothurian feeding mode (C). Circle size indicates the relative contribution of each cell to the total Chi-squared score. To the right are the residual values. The signs of the residual values indicate the positive or negative association between rows (species, echinoderm groups and holothurians) and columns (color of fibers). Positive residuals are blue (and represented by blue circles) and indicate a positive association between the corresponding row and the column variables. Negative residuals are red and imply a negative association between the pair of variables. Data obtained from deep-sea benthic invertebrates sampled from the Antarctic continental shelf and deposited in the Universidade de São Paulo (ColBIO) and University of Hawai'i at Mānoa collections.

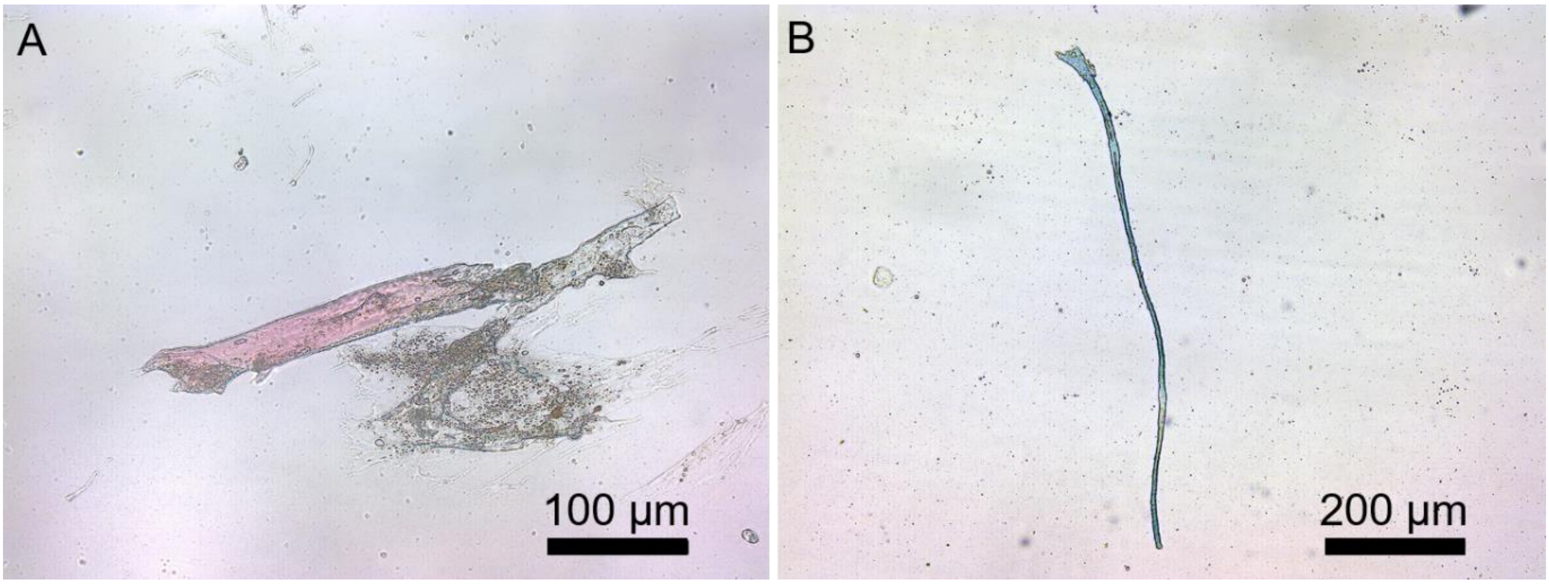

Figure S3. Microplastics ingested by deep-sea benthic invertebrates sampled south-southwest of Anvers Island, western Antarctic Peninsula, in June and October 2000, respectively. A. Red polyamide fiber found in a sea cucumber *Scotoplanes globosa* (Echinodermata: Holothuroidea) individual. B. Blue polyamide and polyester blend fiber found in a sea cucumber *Molpadia violacea* (Echinodermata: Holothuroidea) individual. Organisms are deposited in the University of Hawai'i at Mānoa collection.

Table S3. GLMs conducted in our study to test for the influence of organism size and feeding mode on the number of fibers ingested by deep-sea benthic invertebrates sampled from the Antarctic continental shelf and deposited in the Universidade de São Paulo (ColBIO) and University of Hawai'i at Mānoa collections. Single- and multipredictor models are included, with and without outliers. Details indicate excluded outliers and asterisks denote statistical significance.

Model	Parameters	Coefficients	df	*F* test	p-value	Details
1. Fibers ~ log(length)	log(length)	-0.5229	1	6.742	0.0008*	All individuals included
	Intercept	1.2793	-	-	-	
	Residuals	-	166	-	-	
2. Fibers ~ log(length) + feeding	log(length)	0.0933	1	7.350	0.007*	All individuals included
	Feeding mode	-	3	5.988	0.00067*	
	F. mode (deposit)	0.9949	-	-	0.0382*	
	F. mode (predator)	-0.8009	-	-	0.1535	
	F. mode (scavenger)	-1.5981	-	-	0.059*	
	F. mode (suspension)	1.2080	-	-	0.0149*	
	Residuals	-	163	-	-	
3. Fibers ~ feeding	Feeding mode	-	3	8.157	0.00004*	All individuals included
	F. mode (deposit)	0.9635	-	-	0.043*	
	F. mode (predator)	-0.7687	-	-	0.1681	
	F. mode (scavenger)	-1.2238	-	-	0.0062*	
	F. mode (suspension)	1.1125	-	-	0.020*	
	Residuals	-	165	-	-	
4. Fibers ~ log(length)	log(length)	-0.3691	1	3.554	0.027*	Without the outliers
	Intercept	0.6077	-	-	-	
	Residuals	-	164	-	-	
5. Fibers ~ log(length) + feeding	log(length)	0.1826	1	3.981	0.0477*	Without the outliers
	Feeding mode	-	3	7.575	0.00009*	
	F. mode (deposit)	0.9166	-	-	0.058*	
	F. mode (predator)	-0.8542	-	-	0.1281	
	F. mode (scavenger)	-2.225	-	-	0.010*	
	F. mode (suspension)	1.1834	-	-	0.018*	
	Residuals	-	161	-	-	
6. Fibers ~ feeding	Feeding mode	-	3	8.011	0.00005*	Without the outliers
	F. mode (deposit)	0.8394	-	-	0.080	
	F. mode (predator)	-0.768	-	-	0.1681	

F. mode (scavenger)	-1.223	-	-	0.0062*
F. mode (suspension)	0.980	-	-	0.043*
Residuals	-	163	-	-

Table S4. Post hoc Tukey's test of the pairwise differences regarding feeding modes for deep-sea benthic invertebrates sampled from the Antarctic continental shelf and deposited in the Universidade de São Paulo (ColBIO) and University of Hawai'i at Mānoa collections. Difference indicates the difference in the observed means of the given pair, the lower and upper confidence interval (CI) of end points and the p-value of the comparison for the given pair of feeding modes. Asterisks denote statistical significance.

Feeding mode pair	Difference	Lower CI	Upper CI	p-value	Presence of outliers
Predator-deposit	-0.6344697	-1.06702335	-0.2019160	0.0011263*	With outliers
Scavenger-deposit	0.4767157	-0.16684832	1.1202797	0.2226111	
Suspension-deposit	0.1239035	-0.37122388	0.6190309	0.9156035	
Scavenger-predator	-0.1577540	-0.77794116	0.4624331	0.9118216	
Suspension-predator	0.7583732	0.29403646	1.2227099	0.0002168*	
Suspension-scavenger	0.6006192	-0.06472325	1.2659616	0.0926256	
Predator-deposit	-0.5444874	-0.91625415	-0.1727207	0.0011524*	Without outliers
Scavenger-deposit	0.3867334	-0.16454220	0.9380090	0.2672244	
Suspension-deposit	0.1029327	-0.32516414	0.5310296	0.9242119	
Scavenger-predator	-0.1577540	-0.68753309	0.3720251	0.8665795	
Suspension-predator	0.6474201	0.24738517	1.0474551	0.0002534*	
Suspension-scavenger	0.4896661	-0.08105472	1.0603870	0.1202682	

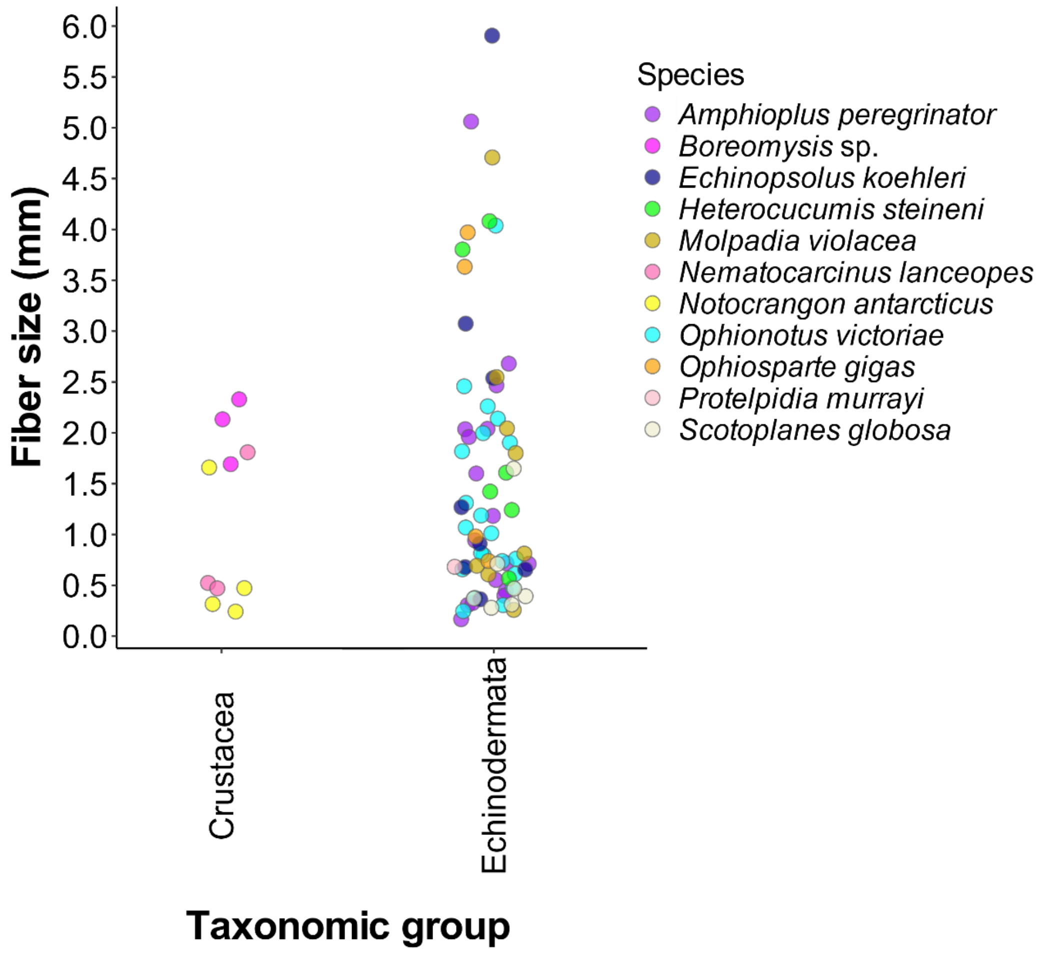

Figure S4. Size of ingested fibers in relation to the two major taxonomic groups surveyed in this study (crustaceans and echinoderms), for deep-sea benthic invertebrates sampled from the Antarctic continental shelf and deposited in the Universidade de São Paulo (ColBIO) and University of Hawai'i at Mānoa collections.
